# The Real Happy Study: Protocol for a Prospective Assessment of the Real-World Effectiveness of the HAPIFED Program—a Healthy APproach to weIght management and Food in Eating Disorders

**DOI:** 10.3390/bs9070072

**Published:** 2019-07-01

**Authors:** Andrea L. Pattinson, Natasha Nassar, Felipe Q. da Luz, Phillipa Hay, Stephen Touyz, Amanda Sainsbury

**Affiliations:** 1 The Boden Institute of Obesity, Nutrition, Exercise & Eating Disorders, Faculty of Medicine and Health, Charles Perkins Centre, The University of Sydney, NSW 2006, Australia; 2 Child Population and Translational Health Research, Children’s Hospital at Westmead Clinical School, Faculty of Medicine and Health, The University of Sydney, NSW 2006, Australia; 3 Translational Health Research Institute (THRI), School of Medicine, Western Sydney University, Penrith, NSW 2751, Australia; 4 School of Psychology, Faculty of Science, The University of Sydney, NSW 2006, Australia

**Keywords:** obesity, overweight, obesity treatment, weight management, diet reducing, eating disorders, binge eating, binge eating disorder treatment, bulimia nervosa, psychotherapy

## Abstract

The prevalence of obesity with comorbid binge eating behaviour is growing at a faster rate than that seen for either obesity or eating disorders as separate conditions. Approximately 6% of the population are affected and they potentially face a lifetime of poor physical and mental health outcomes and an inability to sustain long-term weight loss. Current treatment options are inadequate in that they typically address either obesity or eating disorders exclusively, not the combination of both conditions. By treating one condition without treating the other, relapse is common, and patients are often left disappointed with their lack of weight loss. An integrated approach to treating these individuals is needed to prevent a worsening of the comorbidities associated with excess body weight and eating disorders. A new therapy has recently been developed, named HAPIFED, which addresses both overweight/obesity and comorbid binge eating behaviour with the combination of behavioural weight loss therapy and cognitive behaviour therapy-enhanced (CBT-E). The aim of this paper is to document the protocol for the Real Happy Study, which will evaluate the effectiveness of the HAPIFED program in treating overweight or obesity with comorbid binge-eating behaviour in a real-world setting.

## 1. Background

The prevalence of obesity with comorbid eating disorder behaviours such as binge eating is high and rapidly rising [1]. Data from large, cross-sectional, representative community samples of adults from South Australia, established that in 2015, almost 6% of the population had obesity with binge eating behaviours, an increase of 7.3-fold over the previous 20-year period. This represents a faster rate of growth in prevalence than that seen for either obesity or binge eating behaviour as separate conditions, with respective increases of 2-fold and 5.3-fold over the same time period [1]. This significant population-wide growth is consistent with the prevalence of binge eating reported in individuals with overweight or obesity as described in a number of international publications [2,3]. For instance, a review of 30 studies of individuals with overweight or obesity reported that between 16% and 51.6% engaged in binge eating behaviour [2]. A higher prevalence was reported in a large sample of 45,477 US military veterans with overweight or obesity and seeking assistance for weight management, whereby 78% of participants self-reported binge eating with associated feelings of loss of control, at least twice a week [3].

Eating disorders are defined in the American Psychiatric Association’s Diagnostic and Statistical Manual of Mental Disorders—5th Edition (DSM-5) and include a range of conditions, such as anorexia nervosa, bulimia nervosa, binge eating disorder, as well as other specified and unspecified feeding and eating disorders (OSFED and UFED). The DSM-5 defines both binge eating disorder and bulimia nervosa as consisting of recurrent episodes of binge eating, characterised by the consumption of an objectively large amount of food during a discrete period of time, such as 2 h. In addition, individuals must experience a sense of loss of control over eating during such episodes, with feelings of being unable to stop eating or control what or how much is being consumed. To meet the DSM-5 criteria, the binge eating behaviour must occur on average at least once a week for a period of three months. Binge eating disorder is further defined by the DSM-5 as causing significant distress and including three or more of the following criteria: eating much more rapidly than normal; eating until feeling uncomfortably full; eating large amounts of food when not feeling physically hungry; eating alone because of feeling embarrassed by how much is being eaten; feeling disgusted with oneself, depressed or very guilty afterward. Bulimia nervosa differs from binge eating disorder in that it is characterised by the presence of recurrent compensatory behaviour in an attempt to prevent weight gain as a result of binge eating. Such behaviour includes self-induced vomiting, misuse of laxatives, diuretics or other medication, fasting, and excessive exercising. Individuals with bulimia nervosa also judge their self-worth to a large degree in terms of their body weight and shape and their ability to control them. As mentioned above, the DSM-5 also lists OSFED as an additional eating disorder diagnosis for feeding or eating behaviours. OSFED is used to define conditions that do not meet the full criteria for any of the specified eating disorder diagnoses, but still cause significant distress to the individual. For example, binge eating disorder of low frequency and/or limited duration would apply to a situation where all of the criteria for binge eating disorder are met, however, at a lower frequency than once a week and/or for less than three months’ duration [4].

There is a growing body of evidence demonstrating that obesity with concurrent eating disorder behaviours is associated with poorer health outcomes than for obesity or eating disorder behaviours individually. In addition to the usual comorbidities that frequently occur with obesity, such as heart disease, stroke, and diabetes [5], a higher prevalence of mood, anxiety, and personality disorders are reported in individuals with obesity and comorbid binge eating [6]. Additionally, higher body mass indices (BMIs) and poorer weight loss outcomes have been reported in treatment-seeking individuals with obesity and binge eating disorder, compared to their counterparts presenting for the same obesity treatment but without a binge eating disorder [7,8].

Despite the apparently greater health needs of individuals with obesity and comorbid binge eating disorders, they are not receiving adequate treatment. Current treatments typically address either obesity or binge eating disorders exclusively, and not the combination of both conditions. Individuals with obesity and comorbid binge eating disorders want and seek to lose weight [9], and generally present for weight loss interventions rather than for treatment of their eating disorder [10,11]. Whilst a number of weight loss interventions may initially be successful in assisting individuals to lose weight [8], weight loss outcomes have been reported to be 50% less in the presence of comorbid binge eating compared to individuals receiving the same treatment and not engaging in binge eating behaviour [12]. This is because the psychopathology that maintains an individual’s tendency to binge is usually not addressed in weight loss interventions [13], meaning that relapses in binge eating behaviour and subsequent weight regain are likely [14,15,16]. 

Although reductions in binge eating behaviour have been demonstrated with behavioural weight loss therapy [17,18], previous research has shown that adjunctive cognitive behaviour therapy (CBT) results in a significantly greater reduction in binge eating than that seen with behavioural weight loss therapy alone [18]. Cognitive behaviour therapy-enhanced (CBT-E) is an alternate form of CBT, differing in that it is designed specifically to treat eating disorders. It is an effective, evidence-based, transdiagnostic treatment [19,20,21] with a core component that addresses the psychopathology that maintains an individual’s eating disorder such as the overvaluation of weight and shape. CBT-E is considered the ‘gold standard’ therapy; however, it does not encourage weight reduction [22,23]. A clinical trial and observations from a clinical setting have shown that when individuals engage in such treatment for their eating disorder behaviours, they are typically not satisfied with the treatment due to their lack of weight loss [9,24].

Weight reduction has historically not been addressed in individuals receiving treatment for their eating disorders, due to a concern that it may exacerbate binge eating or other eating disorder behaviours [25,26]. Recent reviews, however, have shown that clinically supervised dietary obesity treatments do not trigger or worsen binge eating behaviour, albeit most studies have been of short duration (less than 12 months) [27]. A further consideration in treating individuals with binge eating is that by treating only the eating disorder and not addressing the excess body weight, the health implications associated with obesity remain.

While the health consequences of obesity are numerous and well documented [28,29,30,31,32,33], overweight is also associated with a number of adverse health outcomes [34,35,36]. For example, a recent study looking at the relationship between BMI and onset of type 2 diabetes mellitus in individuals aged over 65 years, showed that men and women with a BMI in the overweight range (25–29.9 kg/m^2^) were at a 30% and 10% greater risk, respectively, of developing type 2 diabetes mellitus, compared to individuals with a BMI in the healthy weight range (<25 kg/m^2^) [36]. With losses of 5–10% of body weight, reductions in the incidence of type 2 diabetes mellitus are seen [37,38]. Greater reductions in body weight (i.e., 15 or 20%) have been shown to yield greater improvements in health outcomes, illustrating that the health benefits of weight loss are dose-dependent [39,40,41,42]. Besides reductions in the risk of type 2 diabetes, other health benefits associated with weight loss include improvements in risk factors for cardiovascular disease [38], better sleep [43,44], improved fertility and pregnancy outcomes [45,46], as well as less pain associated with osteoarthritis [47,48,49,50,51]. Improvements in psychological well-being [43,52,53] and quality of life [53,54,55] have also been shown to occur. Therefore, by not addressing excess body weight in the treatment of individuals with overweight or obesity and comorbid binge eating behaviour, such individuals are being denied the physical and mental health benefits that generally accompany weight loss. 

Given the lack of comprehensive treatment options available for individuals with overweight or obesity and comorbid binge eating, there is a high risk that such individuals may ‘slip through the cracks,’ with their ongoing binge eating potentially contributing to further weight gain and a worsening of the comorbidities associated with excess weight and eating disorders. Therefore, there is a need for therapies that take an integrated approach by addressing both the overweight or obesity and eating disorder components. 

A new program, entitled HAPIFED (A **H**ealthy **AP**proach to we**I**ght management and **F**ood in **E**ating **D**isorders) has recently been developed which aims to bridge the gaps in currently available therapies by combining both behavioural weight loss therapy to address overweight or obesity with CBT-E, to treat the eating disorder psychopathology [56]. However, since this is a new and novel program, evaluation is required.

A randomised controlled trial comparing the HAPIFED program with CBT-E, which—as mentioned above—has no weight loss component, is currently underway in Brazil [57]. To complement the findings from that randomised controlled trial and to develop strategies to enhance translation of the HAPIFED program into wider-scale clinical practice, we have chosen to measure effectiveness in a real-world setting. The eligibility criteria for real-world studies are less rigid than those for randomised controlled trials, allowing for a study population that is more representative of the population at large. This enables the external validity of results to be established, making them more applicable and generalizable to clinical practice [58]. Moreover, because the implementation of an intervention in a real-world study is more flexible than in a randomised controlled trial, the HAPIFED program may be modified to suit a real-world setting, with these modifications noted and used to further enhance the program for clinical practice [58]. As such, the information derived from the real-world assessment of the HAPIFED program will be complementary to that derived from the randomised controlled trial and provide further insight into how individuals with eating disorders might best be treated with this new program.

The aim of this paper is thus to outline the protocol for the Real Happy Study: a real-world assessment of the HAPIFED program. 

## 2. Methods

### 2.1. Clinical Hypothesis

We hypothesize that the HAPIFED program for the treatment of individuals with overweight or obesity and comorbid binge eating behaviours will result in weight loss and a reduction in binge eating and eating disorder symptoms, as assessed with the global score on the eating disorder examination questionnaire (EDE-Q).

### 2.2. Study Design

This will be a prospective study of the real-world effectiveness of the HAPIFED program for the treatment of individuals with overweight or obesity and who identify as having comorbid disordered binge eating behaviours that are characteristic of comorbid binge eating disorder, bulimia nervosa, or OSFED with recurrent binge eating.

### 2.3. Program

The HAPIFED program combines behavioural weight loss therapy to address overweight or obesity, with CBT-E to treat the eating disorder psychopathology. The program is designed to be led by a psychologist or therapist and conducted in groups of up to 12 individuals. It is comprised of up to 30 sessions, conducted on a weekly basis over a 6-month period. Each session is expected to last for approximately 90 min. Since this study is evaluating the real-world effectiveness of HAPIFED, the implementation of the program is at the therapist’s discretion and may be shortened or adapted in other ways to suit the individual circumstances of the therapist, participants, facilities or environments in which it is being conducted. Any deviations to the prescribed program will be captured in the evaluation of the interventions, as described in Section 2.9 *(Process Evaluation)*. 

HAPIFED focuses heavily on the self-monitoring of eating behaviour. For instance, it requires participants to record the timing of eating or drinking anything containing energy (kilojoules or kilocalories), and the emotional, psychological, social, or environmental circumstances that influence their consumption. Appetite awareness is monitored by ratings for hunger and satisfaction, which are intended to be recorded before and after each time a participant consumes anything containing energy. The recorded variables and their management are all addressed throughout the course of the program. 

The HAPIFED program is implemented in 3 stages, an overview of which is detailed below as an excerpt from the published manual. Full details of the HAPIFED program and manual are published elsewhere [56] (HAPIFED: A **H**ealthy **AP**proach to we**I**ght management and **F**ood in **E**ating **D**isorders 30 Group Session Program Manual © da Luz, Swinbourne, Hay, Touyz, Palavras, Claudino, Sainsbury 2017). 

**Stage 1 (Sessions 1 to 7): Introduction and Psychoeducation**

- Engage the participant in the program and in the process of change.
- Provide relevant psychoeducation about obesity, weight loss, and eating disorders.
- Provide initial education about nutrition and physical activity.
- Introduce proactive problem-solving techniques.
- Explain the difference between healthy versus disordered eating and exercise habits.

**Stage 2 (Session 8 to 26): Core Interventions**

- Address changes in eating related to events and mood.
- Encourage participants to improve their interpersonal functioning and fortify their supportive relationships.
- Guide participants through mindfulness and relaxation exercises.
- Address dissatisfaction with body image and weight.
- Address unhealthy body checking, comparison, and avoidance.
- Encourage participants to engage in self-nurturing activities.
- Improve participants’ knowledge regarding nutrition and physical activity and motivate participants to continue engagement with behavioural weight loss strategies.
- Help participants to identify any existing or emerging barriers to change.
- Help participants to personalize their HAPIFED formulation and adjust interventions according to their individualized formulation.
- Use cognitive therapy and behavioural experiments in order to help participants to restructure any unhelpful thinking styles they may have and reduce their disordered eating behaviours.
- Encourage participants to engage in activities that enhance positive self-evaluation and are not related to shape, weight, or eating.

**Stage 3 (Sessions 27 to 30): Relapse Prevention**

- Help participants to think about strategies to maintain healthy eating and healthy physical activity levels after program completion.
- Encourage the maintenance of any improvements in eating disorder symptoms.
- Help participants to develop a detailed post-program management plan.

### 2.4. Study Intervention

The intervention under study is the HAPIFED program; however, it is not being provided as part of the current study. Rather, HAPIFED is being provided by the therapist delivering the program as part of their practice. Data will be collected using online questionnaires, from individuals who are undertaking the HAPIFED program as part of their prescribed healthcare, as well as from their therapists.

### 2.5. Duration

The total duration of the study is 156 weeks (36 months). Participants will take part in the HAPIFED program for up to 30 weeks (approximately 7 months), and data will continue to be collected for a total of 156 weeks (36 months) from commencement of therapy, to determine long-term effectiveness (Table 1).

### 2.6. Study Participants and Eligibility Criteria

Individuals with overweight or obesity who identify as having disordered binge eating behaviours that are characteristic of binge eating disorder, bulimia nervosa, or OSFED with recurrent binge eating, will be eligible for the study.

#### 2.6.1. Inclusion Criteria

- Male or female aged 18 years and over;
- Able to read, write and communicate in English and be willing to provide written, informed consent;
- A BMI of ≥25 kg/m^2^. This criterion includes individuals with obesity (BMI ≥ 30 kg/m^2^), as well as those with a BMI in the overweight range (25–29.9 kg/m^2^). A BMI of ≥25 kg/m^2^ is associated with an increased risk of disease [34,35]. Weight loss offers numerous health benefits to such individuals [37,38,43,44,45,47,48,49,50,52,53,54,55] and may indeed serve as a preventative measure in ensuring that an individual’s weight trajectory does not increase from the overweight to the obese range, as is often seen in those with binge eating disorder [59]. Individuals with a BMI of ≥25 kg/m^2^ often want and seek to lose weight [9,24]. It is therefore appropriate to allow such individuals to enter the study if they wish;
- A global score on the EDE-Q that is greater than 1 standard deviation (1.25) above the Australian community norm of 1.52 (i.e., above 2.77) [60];
- Willing to undertake the HAPIFED program of up to 30 weeks (approximately 7 months) in duration involving behavioural weight loss therapy and CBT-E, and to attend sessions regularly (i.e., not miss more than 3 sessions over the course of the program);
- Have access to a computer, the Internet and an e-mail account and be willing to complete the online questionnaires up to 156 weeks (36 months) from study entry.

#### 2.6.2. Exclusion Criteria

- Pregnant or breast-feeding, or having a desire to become pregnant during the first 52 weeks of the study;
- Receiving current treatment with a weight loss medication such as, but not limited to, orlistat, phentermine, or liraglutide, or other medication which is known to cause weight loss such as topiramate or lisdexamfetamine, or have received treatment with such medication in the 5 weeks prior to screening, taking into consideration the half-life and subsequent washout period of the medication. The reason for this is to ensure that weight loss as a result of the medication does not contribute to any weight loss during study participation. An additional reason for this exclusion criterion is that the EDE-Q asks about eating behaviour in the previous 4 weeks, and an individual’s responses to the EDE-Q may not be an accurate reflection of their usual behaviour if they have been taking weight loss medication in that time period;
- Undergone bariatric surgery or intragastric balloon insertion in the 24 months prior to screening, due to the fact that weight loss may continue for up to 24 months post-procedure [61,62];
- Greater than 3 kg weight change in the 5 weeks preceding screening, to ensure that participants are relatively weight-stable at study entry so that any weight change throughout the study may be confidently attributed to the HAPIFED program without uncertainty that it may be influenced by prior weight changes;
- Diagnosis of a clinical condition, or use of a medical treatment, that interferes with appetite regulation (e.g., Prader–Willi or Cushing’s syndrome, or some steroid treatments). The reason for this criterion is that the behavioural weight loss intervention in HAPIFED requires participants to eat according to appetite, but this is likely to result in weight gain for people with conditions or who are using treatments that stimulate appetite.

### 2.7. Study Procedures and Data Collection

#### 2.7.1. Screening/Baseline

Potential participants will be informed of the study by the HAPIFED therapist and provided with a copy of the study brochure listing the selection criteria and instructions on how to take part in the study and complete the online questionnaires. Interested participants will be directed to a secure online webpage that contains the ‘Information for Participants,’ an online screening questionnaire, and a consent form.

Following consent, participants will complete the online baseline questionnaires. There will be no time limit for completion of the online questionnaires; however, it is expected to take approximately 40 min and will include the following components:

- Height;
- Weight;
- Demographic Questionnaire: gender, marital status, ethnicity, occupation, education;
- Commonwealth Scientific and Industrial Research Organisation (CSIRO) Healthy Diet Score Survey [63];
- Eating Disorder Examination Questionnaire (EDE-Q) [64,65];
- Depression, Anxiety, and Stress Scale with 21 Items (DASS-21) [66];
- Short-Form Health Survey with 12 Items, Version 2 (SF-12v2) [67];
- Cost-Effectiveness questionnaire, which includes information about medication use (current medication including name of drug, total daily dose, dosing frequency, and indication).

#### 2.7.2. Follow-Up

Participants will be followed up at 30 weeks (approximately 7 months) after commencement of the HAPIFED program, by which stage it is expected to be completed, and then at 52 weeks (12 months), 104 weeks (24 months), and 156 weeks (36 months), following commencement of the program to determine long-term effectiveness (Table 1). As for the baseline questionnaires, participants will be sent an e-mail with instructions and an embedded web link to complete the same online questionnaires as completed at baseline, but without the question about height or the Demographic Questionnaire. They will also be required to complete the Client Satisfaction Questionnaire (CSQ-8) [68] and the Process Evaluation Questionnaire for Participants at week 30 (month 7).

All nonrespondents will be identified and sent up to two reminder emails for each data collection time point.

### 2.8. Outcome Measures

#### 2.8.1. Primary Outcomes

The co-primary outcomes are as follows:

(1) The proportion of participants that have lost ≥5% of their baseline body weight at 52 weeks (12 months) after program commencement.
(2) The proportion of participants that have a global score on the EDE-Q that is less than 1 standard deviation (1.25) above the Australian community norm of 1.52 (i.e., below 2.77) [60] at 52 weeks (12 months) after program commencement.

Weight loss of ≥5% of body weight is considered clinically significant [69] and is associated with a reduction in the risk of noncommunicable diseases as well as with improvements in overall health and quality of life [43,70,71]. Indeed, the Australian Government’s Therapeutic Goods Administration uses this level as a threshold for evidence of weight loss efficacy for weight loss medications.

The EDE-Q is a widely used and validated self-report version of the diagnostic, interview-based Eating Disorder Examination [72]. This questionnaire will provide a comprehensive assessment of the range and severity of eating disorder psychopathology. The rationale for using the outcome measure of a global score on the EDE-Q that is less than 1 standard deviation above community norms is for the purpose of comparison with previous studies involving CBT-E [22,73,74]. A reduction in the global score on the EDE-Q to a value that is less than 1 standard deviation above community norms is considered a good outcome or ‘remission’ [22], and indeed, the majority of studies conducted with CBT-E used this threshold as a measure of efficacy [22,73,74].

#### 2.8.2. Secondary Outcomes

The secondary outcomes are the change in the following parameters between baseline and follow-up at week 30 (approximately 7 months) and at 12, 24, and 36 months:

- Anthropometric parameters:
  ○ absolute weight;
  ○ weight as a percentage of baseline body weight;
  ○ BMI;
  ○ the proportion of participants who achieve ≥5% loss of baseline body weight;
  ○ the difference in the effect of HAPIFED on anthropometric parameters for people with different classifications of overweight or obesity at baseline (Overweight = BMI of 25–29.9 kg/m^2^; Class 1 Obesity = BMI of 30–34.9 kg/m^2^; Class 2 Obesity = BMI of 35–39.9 kg/m^2^; Class 3 Obesity = BMI of ≥40 kg/m^2^) [75];
- Eating disorder parameters:
  ○ severity of the eating disorder, as assessed using the EDE-Q [65,72];
- Dietary parameters:
  ○ nutritional quality of food choices, including the intake of fruit and vegetables, as assessed using the CSIRO Healthy Diet Score Survey [63]. The CSIRO Healthy Diet Score Survey is a scientifically validated survey that assesses an individual’s diet against Australia’s healthy eating guidelines [76];
- Psychosocial parameters:
  ○ depression, anxiety, and stress, as assessed using the DASS-21 [66]. The DASS-21 is a self-report scale with 21 items used to assess symptoms of depression, anxiety and stress;
- Quality of life as assessed using the SF-12v2 [67]. The SF-12v2 is a self-report scale with 12 items used to assess mental and physical functioning and overall health-related quality of life;
- Economic parameters:
  ○ cost-effectiveness of the HAPIFED program as assessed by the use of a questionnaire to determine health-care utilization, medication use, and days off work.

### 2.9. Process Evaluation

Participants and therapists will be asked to complete an online Process Evaluation Questionnaire at the completion of the program (Table 1). 

The Process Evaluation Questionnaires will be used to collect information on the implementation and acceptability of the program to both the participant and the therapist. This information will be used in conjunction with the CSQ-8 [68] to evaluate the HAPIFED program in clinical practice, and to inform strategies to enhance translation of the findings from this study into wider-scale clinical practice. 

### 2.10. Sample Size

Based on the Australian government’s recommendation for obesity treatments that at least 50% of participants in a treatment group must achieve a weight loss of at least 5% of initial body weight to demonstrate a clinically significant benefit [69], and the HAPIFED pilot results where 50% of the sample had an EDE-Q score of less than one standard deviation (1.25) above the community normative score of 1.52 (i.e., below 2.77) [56,60] at the end of treatment (whereas all had a score above 2.77 before treatment), we estimated that a sample size of at least 164 participants (with 5% significance level and power of 80%) is required. To allow for an up to 50% attrition rate as that found in the pilot study [56], we inflated the sample size to 246 to ensure complete data. 

### 2.11. Statistical Analysis

Paired t-tests will be used to compare continuous baseline data with follow-up data for normally distributed variables, or Wilcoxon signed-rank tests for non-normally distributed variables. McNemar’s tests will be used to analyse categorical data. Multivariable logistic regression analysis with repeated measures will be conducted to assess dichotomous outcomes (e.g., ≥5% weight loss versus not), taking into account important covariates and paired data. Statistical significance will be demonstrated with a *p*-value of 0.05 and Cohen’s d will be used to indicate effect size. Analysis will be conducted for the complete cohort (intention-to-treat), as well as for those that complete the HAPIFED program. Median number of sessions, sociodemographic factors, outcomes, and scores will be compared between completers and noncompleters. Cost-effectiveness will be assessed using the quality of life data, health service utilization costs (determined from the self-report cost-effectiveness questionnaire and costed using the Medicare Benefits Schedule), and medication costs (using the Pharmaceutical Benefits Scheme), as previously published [77,78].

### 2.12. Ethics Approval and Trial Registration

The study is approved by the Sydney Local Health District, Royal Prince Alfred Hospital Human Research Ethics Committee for Protocol No X17-0088. The study was prospectively registered, on 14 July 2017, with the Australian New Zealand Clinical Trials Registry (ACTRN12617001020370). 

## 3. Discussion

The HAPIFED program offers a unique approach to treating overweight or obesity with comorbid binge eating behaviours by combining behavioural weight loss therapy with CBT-E. The aims of the program are: (1) To promote strategies for healthy weight management by eating nutritious foods according to physical hunger and satisfaction, combined with healthy physical activity; (2) To reduce binge eating and compensatory behaviour(s) such as excessive (unhealthy or compulsive) physical activity, fasting, self-induced vomiting or misuse of laxatives or diuretics; and (3) To promote healthy self-evaluation that is not dominated by concerns related to food, eating, weight, or shape [56].

This study seeks to provide evidence of the “real-world” effectiveness of the HAPIFED program. The pragmatic methodology is conducive to generating results that are applicable to clinical practice and which may be used to enhance the acceptability of the program. It is hypothesized that the HAPIFED program will achieve positive outcomes in the form of weight loss, weight loss maintenance, and eating disorder treatment, and thus improve the physical and mental health of individuals.

## Figures and Tables

**Table 1:** Schedule of Assessments.

	ASSESSMENT	SCREENING/BASELINE	WEEK 30 (MONTH 7)	WEEK 52 (MONTH 12)	WEEK 104 (MONTH 24)	WEEK 156 (MONTH 36)
		WEEK 0 (MONTH 0)				
Conducted by Participants (online)	Screening/Eligibility	✓				
	Written Informed Consent	✓				
	Height	✓				
	Weight	✓	✓	✓	✓	✓
	Demographic Questionnaire	✓				
	CSIRO * Healthy Diet Score Survey	✓	✓	✓	✓	✓
	Eating Disorder Examination Questionnaire (EDE-Q)	✓	✓	✓	✓	✓
	Depression, Anxiety and Stress Scale with 21 Items (DASS-21)	✓	✓	✓	✓	✓
	Short-Form Health Survey with 12 Items, Version 2 (SF-12v2)	✓	✓	✓	✓	✓
	Cost-Effectiveness Questionnaire	✓	✓	✓	✓	✓
	Client Satisfaction Questionnaire (CSQ-8)		✓			
	Process Evaluation Questionnaire for Participants		✓			
Conducted by Therapists (online)	Process Evaluation Questionnaire for Therapists		✓			

* CSIRO, Commonwealth Scientific and Industrial Research Organisation.

## Abbreviations

BMI	Body Mass Index
CBT	Cognitive Behaviour Therapy
CBT-E	Cognitive Behaviour Therapy-Enhanced
CSIRO	Commonwealth Scientific and Industrial Research Organisation
CSQ-8	Client Satisfaction Questionnaire
DASS-21	Depression, Anxiety and Stress Scale with 21 Items
DSM-5	Diagnostic and Statistical Manual of Mental Disorders-5th Edition
EDE-Q	Eating Disorder Examination Questionnaire
HAPIFED	A **H**ealthy **AP**proach to We**I**ght Management and **F**ood in **E**ating **D**isorders
SF-12v2	Short-Form Health Survey with 12 Items, Version 2
